# A122 CHARACTERIZATION OF COLORECTAL NEOPLASIA IN PEOPLE WITH LYNCH SYNDROME

**DOI:** 10.1093/jcag/gwae059.122

**Published:** 2025-02-10

**Authors:** K Al-Bayati, K Brar, H Rothenmund, B Chordiker, J Stone, C Kim, H Singh

**Affiliations:** Department of Internal Medicine, Max Rady College of Medicine, University of Manitoba, Winnipeg, MB, Canada; Department of Internal Medicine, Max Rady College of Medicine, University of Manitoba, Winnipeg, MB, Canada; Department of Biochemistry and Medical Genetics, Max Rady College of Medicine, University of Manitoba, Winnipeg, MB, Canada; Department of Biochemistry and Medical Genetics, Max Rady College of Medicine, University of Manitoba, Winnipeg, MB, Canada; Department of Internal Medicine, Max Rady College of Medicine, University of Manitoba, Winnipeg, MB, Canada; Department of Internal Medicine, Max Rady College of Medicine, University of Manitoba, Winnipeg, MB, Canada; Department of Internal Medicine, Max Rady College of Medicine, University of Manitoba, Winnipeg, MB, Canada

## Abstract

**Background:**

Lynch syndrome (LS) is an autosomal dominant condition that is associated with germline variants in the MLH1, MSH2, MSH6, or PMS2 genes or a deletion in the EpCAM gene which inactivates MSH2. LS is the most common cause of hereditary colorectal cancer (CRC), accounting for 3% of all new diagnoses. In 2017, Manitoba implemented an initiative where all CRC biopsy and surgical specimens for patients ≤ 70 years of age are screened for LS through mismatch repair immunochemistry. If confirmed to have LS, cascade testing is offered, and systematic evidence-based screening is recommended. Recent hypotheses suggest alternative pathways of development of CRC in LS, with variation by affected gene. Guidelines recommend delaying onset of screening colonoscopy to age 30-35 for those with MSH6 and PMS2 mutations. However, there are limited contemporary data on pre-CRC lesions in LS, including any differences according to impacted gene. Lower rate of CRC in young persons with MSH6/PMS2 may be related to removal of pre-CRC lesions

**Aims:**

The objective of this study is to characterize colorectal lesions among a population-based cohort of LS individuals

**Methods:**

The database of individuals with pathogenic/likely pathogenic (P/LP) LS gene variants in Manitoba is augmented with colonoscopy data and pathological findings. We report descriptive statistics to describe the neoplastic lesions (pre-CRC lesions and CRC) in those with LS with data stratified by gene type. Prevalence estimates among individuals with LS without CRC amongst those with advanced CRC precursor lesions was reported. Advanced CRC precursor lesions are defined as either ≥1 cm in size, villous/tubulovillous histology, high grade dysplasia, or serrated polyps with dysplasia

**Results:**

A total of 482 people with LS were analysed (33.9% males). 198 individuals with LS had documented CRC, of which 28.3% were in the cecum. Prevalence of advanced CRC precursors was 9/81 in people with MSH6 mutation, 18/102 with MLH1 mutation, 16/112 with PMS2 mutation, and 30/136 with MSH2 mutation. Median age at diagnosis of advanced CRC precursors was 56-years with average polyp size of 16.5 mm. 1/8 individuals under the age of 35 years old at the time of the colonoscopy in the MSH6 group had advanced CRC precursors, compared to 1/25 of individuals in the PMS2 mutation group. There were no CRC diagnosed below the age of 35 in the PMS2 and MSH6 groups

**Conclusions:**

This population-based study is characterizing neoplastic lesions in individuals with LS highlighting the distribution of these lesions according to gene variants. The data analysis thus far raises concerns on the recent guidelines recommendations to increase the age of onset of colonoscopy surveillance to 35 years old in those with PMS2/MSH6 mutation; those recommendations did not consider the effect of removal of CRC precursors on CRC occurrence

**Funding Agencies:**

None

